# Comparisons of objective and subjective refraction with and without cycloplegia using binocular wavefront optometer with autorefraction and retinoscopy in school-age children

**DOI:** 10.1007/s00417-022-05936-8

**Published:** 2022-12-17

**Authors:** Yadi Lei, Xun Chen, Mingrui Cheng, Boliang Li, Yinjie Jiang, Yilin Xu, Xiaoying Wang

**Affiliations:** 1 Fudan University Eye Ear Nose and Throat Hospital, Shanghai, China; 2 National Health Commission Key Lab of Myopia, Fudan University, Shanghai, China; 3 Shanghai Research Center of Ophthalmology and Optometry, Shanghai, China; 4 Shanghai, China

**Keywords:** Refraction, Retinoscopy, Cycloplegia, Children, Adaptive optics, Binocular wavefront optometer

## Abstract

**Purpose:**

To compare school-age children’s objective and subjective refraction using a binocular wavefront optometer (BWFOM) with autorefraction and retinoscopy before and after cycloplegia.

**Methods:**

Eighty-six eyes from 86 children (6–15 years old) were enrolled in this cross-sectional study. BWFOM objective and subjective refractions were compared with autorefraction and retinoscopy under cycloplegia. BWFOM refraction was evaluated before and after cycloplegia. Measurements were compared using a paired *t*-test; agreement was assessed using Bland–Altman plots.

**Results:**

Under cycloplegia, the sphere, spherical equivalence, and J45 were significantly more negative on BWFOM objective refraction than autorefraction (− 1.39 ± 2.20 D vs. − 1.28 ± 2.23 D, *P* = 0.003; − 1.84 ± 2.38 D vs. − 1.72 ± 2.43 D, *P* = 0.001; − 0.02 ± 0.17 D vs. 0.03 ± 0.21 D, *P* = 0.004). The subjective sphere of BWFOM was less myopic, and the cylinder and the J45 were more negative than those with retinoscopy (− 1.17 ± 2.09 D vs. − 1.25 ± 2.20 D, *P* = 0.02; − 0.91 ± 0.92 D vs. − 0.76 ± 0.92 D, *P* < 0.001; − 0.01 ± 0.15 D vs. 0.03 ± 0.21 D, *P* = 0.028). For both BWFOM objective and subjective refraction, sphere and spherical equivalence with noncycloplegia were more myopic than those with cycloplegia (objective: − 1.76 ± 2.10 D vs. − 1.39 ± 2.20 D, − 2.21 ± 2.30 D vs. − 1.84 ± 2.38 D, *P* < 0.001; subjective: − 1.57 ± 1.92 D vs. − 1.17 ± 2.09 D, − 2.01 ± 2.13 D vs. − 1.62 ± 2.27 D, *P* < 0.001). Bland–Altman plots showed good agreement in spherical equivalence between BWFOM objective refraction and autorefraction (mean difference = 0.12 D, 95% confidence interval [CI] − 0.52 to 0.76), subjective refraction with retinoscopy (mean difference =  − 0.01 D, 95% CI − 0.65 to 0.64), and BWFOM refractions with or without cycloplegia (objective: mean difference =  − 0.37 D, 95% CI − 1.31 to 0.57; subjective: mean difference =  − 0.39 D, 95% CI − 1.30 to 0.51). The time cost by BWFOM was significantly less than the total time of autorefraction and retinoscopy (264.88 ± 90.67 s vs. 315.89 ± 95.31 s, *P* < 0.001).

**Conclusion:**

BWFOM is a new device that realizes both objective and subjective refraction. For children’s refractive errors, it is more convenient and quicker to obtain the proper prescription at a 0.05-D interval, and it is more accurate than autorefraction and retinoscopy under cycloplegia. 
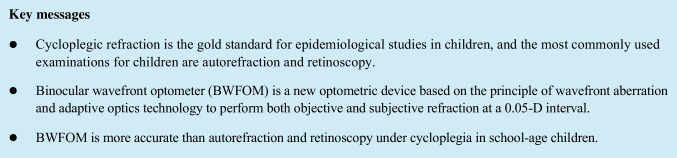

## Introduction

Refractive error is one of the most common causes of visual impairment in children and adults and has become a public health priority as its prevalence increases worldwide [1]. Myopia is the most common type of refractive error. One study predicted that, by 2050, there will be 4758 million people with myopia, almost 49.8% of the world population, and 938 million people with high myopia [2]. The focus of myopia prevention and control should be to reduce the prevalence of myopia and prevent the irreversible damage to uncorrected refractive errors and vision associated with high myopia [3]. Therefore, it is important to detect refractive errors and intervene at an early stage.

Refractive errors in children are not easily detected; therefore, timely examination and accurate refraction are key points [4]. Current research has shown that cycloplegic refraction is the gold standard for epidemiological studies in children, even for adults under the age of 50 [5]. The most commonly used examinations for children are autorefraction and retinoscopy. In particular, cycloplegic retinoscopy is considered the gold standard of refraction in children due to its high degree of accommodation [6]. However, it requires a professionally trained individual and a certain level of cooperation from the children. Moreover, it must be performed in a dark room. Autorefractors are widely used in hospitals because they are fast and easy to use; however, they may be insufficiently accurate for proper glass prescriptions [7].

Recently, new technologies and devices for optometry have emerged. Binocular wavefront optometer (BWFOM, Aizhitong Medical Technology Co., Ltd, Zhejiang, China) is a new device that enables both objective and subjective refraction and can be used in a bright room. It uses built-in optical scales, wavefront aberration, and adaptive optics technology. BWFOM can obtain optometric prescriptions at a 0.05-D interval and can provide glasses with a 0.05-D interval lens. Compared with the traditional 0.25-D interval optometry method, the BWFOM 0.05-D interval appears to be more precise, allowing for a fast and accurate red-green balance. BWFOM uses cloud storage technology and big data analysis to form a personal refractive profile and store it permanently. Therefore, individuals are required to bind the applet in advance to quickly obtain a prescription after the examination. BWFOM also can print optometry results directly when there is no internet. Objective refraction is achieved by using the Hartmann–Shack wavefront measurement technique. Optical modulators are built into the device to achieve optical defocus and astigmatism regulation as well as achieve greater precision in optical correction.

This study aimed to compare the objective and subjective refraction of school-age children before and after cycloplegia using BWFOM with autorefraction and retinoscopy to assess the accuracy of the new refractive techniques.

## Participants and methods

### Participants

This cross-sectional study included 86 eyes from 86 children aged between 6 and 15 years [8]. All participants were required to have clear ocular media and were excluded if they had ocular pathologies, including nystagmus, amblyopia, strabismus, any disease that can cause media opacity, congenital or acquired optic nerve disease, retinal disease, glaucoma, previous ophthalmic surgery, history of contact lens wear, and an inability to finish the examination. This study adhered to the tenets of the Declaration of Helsinki and was approved by the ethics committee of our hospital. Informed consent was obtained from the parents of all participants.

#### BWFOM

**Fig. 1:**
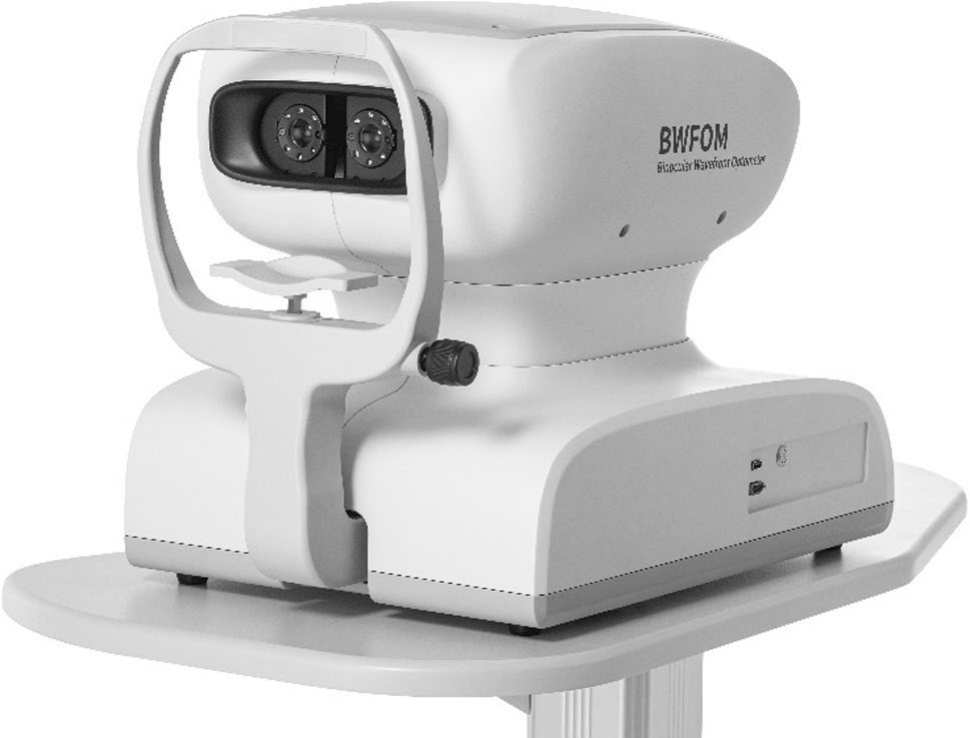
The appearance of binocular wavefront optometer (BWFOM)

BWFOM is a newly introduced automated optometry device with both objective and subjective refraction functions (Fig. 1). This allows for convenient and accurate refraction in a relatively short time. The physician used the software on a companion tablet to guide the examined person to complete the examination. Since all the data are saved on the internet, physicians and the examined person can obtain optometric prescriptions directly on cell phones, tablets, or other mobile devices. Objective refraction utilizes wavefront aberration and adaptive optics technology, which is measured using a Hartmann–Shack wavefront sensor, and separates higher- from lower-order aberrations, converting the lower-order aberration data into objective refractive values that are used as the starting point for subjective refraction [9, 10]. The principle of subjective refraction is similar to that of phoropters, except that it uses a set of optical modulators to simulate optical lenses, such as spherical and cylindrical lenses. For the examiner, subjective refraction can be obtained by simply making manual adjustments on the operating interface based on patient feedback. The modulator receives the command and moves back and forth on the optical axis until the examined person is satisfied with the refraction, with an accuracy of up to 0.01 D. Measurements have shown that most people are sensitive to smaller dioptric changes [11, 12]; therefore, BWFOM improves the precision of refraction, enabling the spherical interval change from 0.25 to 0.05 D through this modulator’s movement, and results in a 0.05-D interval optometric prescription that can be directly obtained.

### Examinations

All participants underwent basic ophthalmic examinations to confirm their eligibility for inclusion on weekend mornings. Objective and subjective refraction were then performed using BWFOM in both eyes and without cycloplegia, with room illumination of 300 lx. Subsequently, cycloplegia was induced using 0.5% tropicamide phenylephrine eye drops (Santen Oy, Tampere, Finland) administered every 5 min for a total of five times, and waiting at least 30 min after the fifth drip [13]. Cycloplegia and pupil dilation were evaluated by an experienced ophthalmologist. Binocular cycloplegic objective and subjective refraction were repeated using BWFOM. Next, the participants underwent cycloplegic autorefraction using Nidek ARK-1 (Nidek Technologies, Gamagori, Japan) and traditional refraction using retinoscopy. The three methods of refraction were done by three experienced optometrists, and the basic ophthalmic examinations were done by the same ophthalmologist. The sphere, cylinder, axis, and spherical equivalence were recorded during each examination. The examination times for BWFOM, autorefraction, and retinoscopy were also collected.

### Statistical analysis

Statistical analysis was performed using Statistical Package for Social Sciences version 26 (Chicago, IL, USA), and *P* < 0.05 was considered statistically significant. Descriptive statistics included mean and standard deviation. All measurements conformed to a normal distribution and were calculated using a paired two-tailed *t*-test. In cycloplegia, the objective refraction of BWFOM was compared with that of autorefraction, and the subjective refraction of BWFOM was compared with that of retinoscopy. Moreover, the BWFOM results were compared between noncycloplegia and cycloplegia. Bland–Altman plots were used to evaluate the agreement between these refraction results. Pearson correlation analysis was used to assess the correlation between age and time of examination. The main refractive variables in this study were the spherical equivalence (M) and power vectors for astigmatism, axes at 180° and 90° (J0) and at 45° and 135° (J45). Conversion of spherocylindrical refraction values to vector notation was performed using the formulas by Thibos et al. [14]. *M* = *S* + *C*/2; J0 = (− *C*/2)cos(2*α*); J45 = (− *C*/2)sin(2*α*); where *S* is the sphere, *C* the negative cylindrical power, and *α* the cylindrical axis.

## Results

### Participants’ demographics

**Fig. 2:**
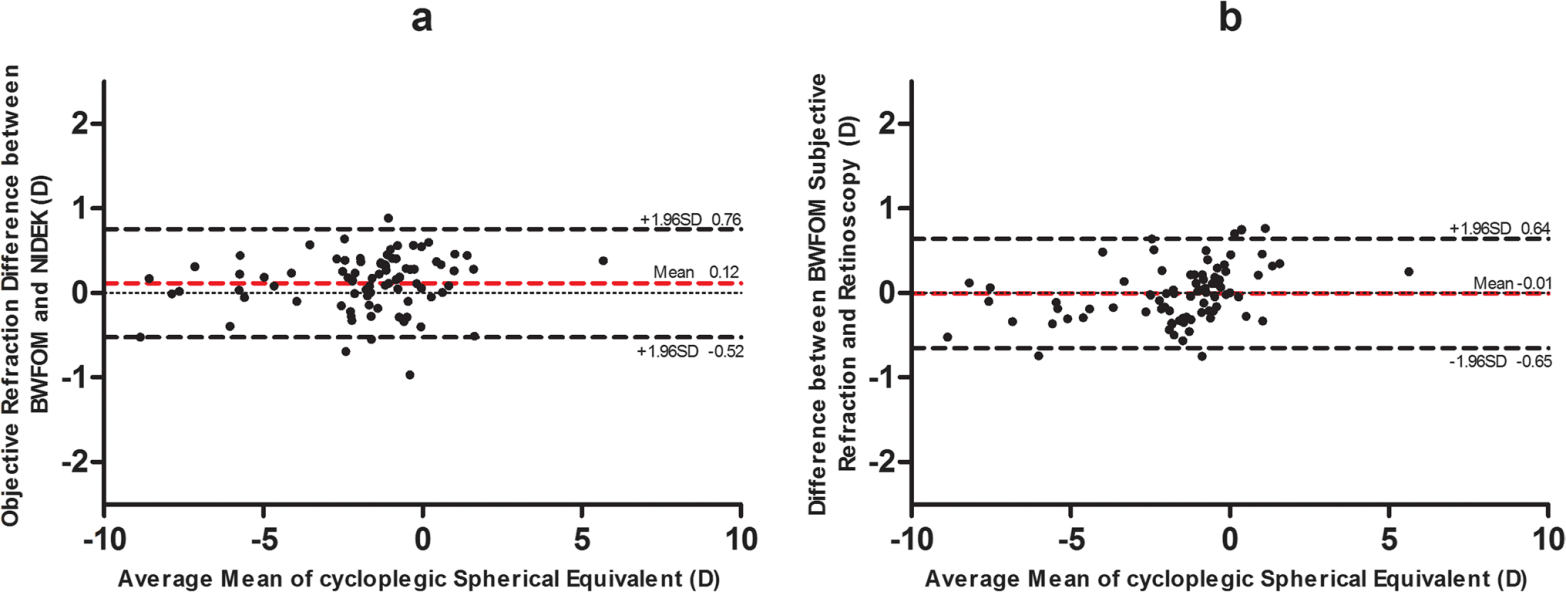
**a** The agreement of BWFOM objective refraction and Nidek autorefraction under cycloplegia. **b** The agreement of BWFOM subjective refraction and retinoscopy under cycloplegia

**Fig. 3:**
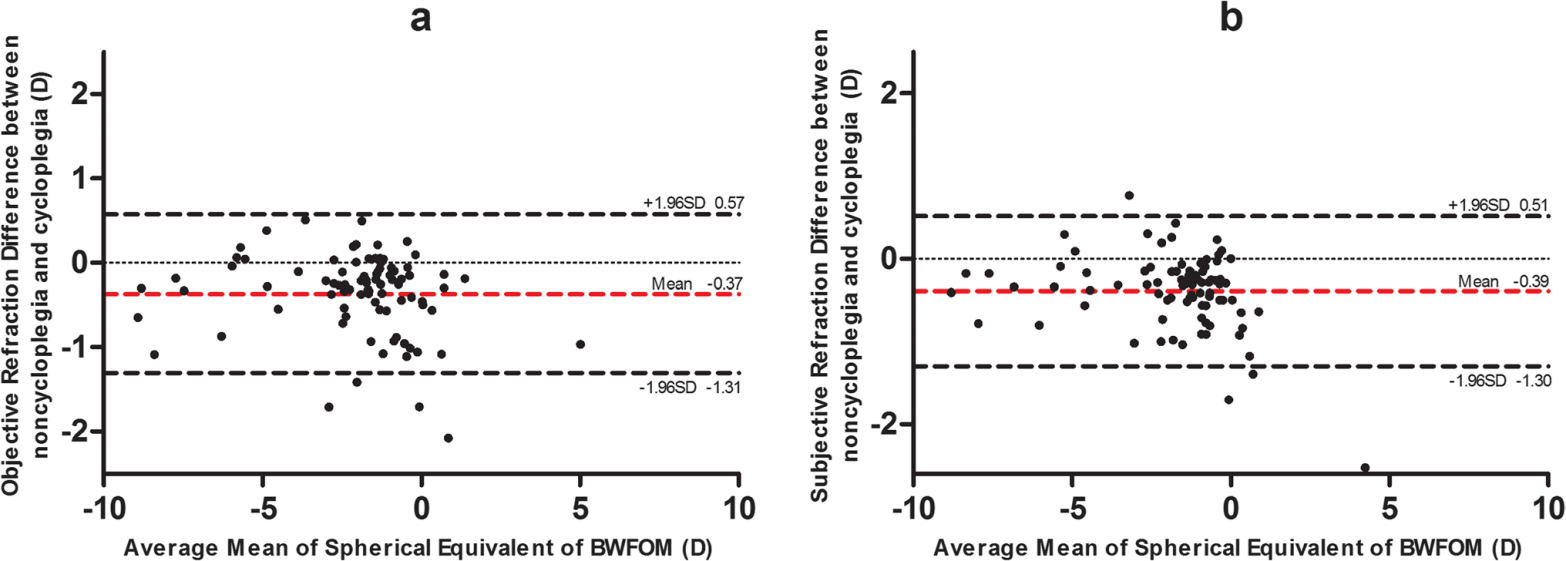
**a** The agreement of objective refraction between noncycloplegia and cycloplegia using the BWFOM. **b** The agreement of subjective refraction between noncycloplegia and cycloplegia using the BWFOM

A total of 86 eyes from 86 patients (male: female = 35:51) were included in this study. Their mean age was 10.20 ± 1.99 (6 to 15) years. Based on cycloplegic retinoscopy of the right eye, the mean sphere was − 1.25 ± 2.20 (− 7.75 to 6.50) D, and the cylinder was − 0.76 ± 0.92 (− 4.00 to 0.00) D. Table 1 shows the mean, standard deviation, and range of refractive power using different methods of refraction under both noncycloplegic and cycloplegic conditions, respectively. Figures 2 and 3 show refraction comparisons between BWFOM and other devices and between noncycloplegia and cycloplegia using Bland–Altman plots.

**Table 1:** Mean, standard deviation, and range of refraction power using different methods of objective and subjective refraction under noncycloplegia and cycloplegia

Parameters	*S* (D)		*C* (D)		*M* (D)		*J*_0_ (D)		*J*45 (D)	
	Mean ± SD	Range	Mean ± SD	Range	Mean ± SD	Range	Mean ± SD	Range	Mean ± SD	Range
Noncycloplegia										
Objective refraction (BWFOM)	− 1.76 ± 2.10	− 8.51 to 5.20	− 0.89 ± 0.93	− 4.70 to − 0.05	− 2.21 ± 2.30	− 9.25 to 4.53	0.37 ± 0.50	− 0.22 to 2.31	0.01 ± 0.17	− 0.47 to 0.64
Subjective refraction (BWFOM)	− 1.57 ± 1.92	− 7.97 to 3.64	− 0.89 ± 0.93	− 4.64 to − 0.05	− 2.01 ± 2.13	− 9.01 to 2.98	0.37 ± 0.50	− 0.22 to 2.28	0.01 ± 0.17	− 0.47 to 0.64
Cycloplegia										
Objective refraction (BWFOM)	− 1.39 ± 2.20	− 8.15 to 6.54	− 0.91 ± 0.93	− 4.02 to 0.23	− 1.84 ± 2.38	− 8.67 to 5.50	0.39 ± 0.47	− 0.13 to 2.00	− 0.02 ± 0.17	− 0.45 to 0.46
Subjective refraction (BWFOM)	− 1.17 ± 2.09	− 7.73 to 6.54	− 0.91 ± 0.92	− 3.96 to − 0.04	− 1.62 ± 2.27	− 8.60 to 5.50	0.35 ± 0.46	− 0.09 to 2.00	− 0.01 ± 0.15	− 0.43 to 0.56
Autorefraction (NIDEK)	− 1.28 ± 2.23	− 8.00 to 6.50	− 0.88 ± 0.92	− 4.00 to 0.00	− 1.72 ± 2.43	− 9.13 to 5.88	0.36 ± 0.50	− 0.40 to 2.00	0.03 ± 0.21	− 0.53 to 0.85
Retinoscopy	− 1.25 ± 2.20	− 7.75 to 6.50	− 0.76 ± 0.92	− 4.00 to 0.00	− 1.63 ± 2.40	− 9.13 to 5.75	0.36 ± 0.49	− 0.39 to 1.97	0.03 ± 0.21	− 0.53 to 0.84

*SD*, standard deviation; *D*, diopter; *S*, sphere; *C*, cylinder; *M* = *S* + *C*/2; *J*_0_ = (− *C*/2)cos(2*α*); *J45* = (− *C*/2)sin(2*α*); *α*, cylindrical axis

### BWFOM versus other devices

For cycloplegic objective refraction of BWFOM, no significant differences were noted in the cylinder (*P* = 0.483) and J0 (*P* = 0.186) and significant differences were noted in the sphere (*P* = 0.003), spherical equivalence (*P* = 0.001), and J45 (*P* = 0.004) compared with NIDEK. For cycloplegic subjective refraction of BWFOM, no significant differences were noted in the spherical equivalence (*P* = 0.887) and J0 (*P* = 0.564) and significant differences were noted in the sphere (*P* = 0.020), cylinder (*P* < 0.001), and J45 (*P* = 0.028) compared with retinoscopy (Table 1). The mean spherical equivalences were − 1.62 ± 2.27 and − 1.63 ± 2.40 D using BWFOM and retinoscopy, respectively. The mean difference of cycloplegic objective refraction between BWFOM and NIDEK was 0.12 ± 0.33 (− 0.97 to 0.89) D, with 84.88% and 45.35% of the eyes within ± 0.50 and ± 0.25 D, respectively. The mean difference of cycloplegic refraction between BWFOM subjective refraction and retinoscopy was − 0.01 ± 0.33 (− 0.75 to 0.77) D, with 88.37% and 55.81% of the eyes within ± 0.50 and ± 0.25 D, respectively.

### Noncycloplegia versus cycloplegia

For objective refraction, no significant differences were observed in the cylinder (*P* = 0.656) and J0 (*P* = 0.457), and J45 (*P* = 0.342) and significant differences were observed in the sphere (*P* < 0.001) and spherical equivalence (*P* < 0.001) between the noncycloplegia and cycloplegia groups using BWFOM. For subjective refraction, no significant differences were noted in the cylinder (*P* = 0.550), J0 (*P* = 0.555), and J45 (*P* = 0.437) and significant differences were noted in the sphere (*P* < 0.001) and spherical equivalence (*P* < 0.001) between the noncycloplegia and cycloplegia groups using BWFOM. The mean difference between the noncycloplegic and cycloplegic objective refraction using BWFOM was − 0.37 ± 0.48 (− 2.08 to 0.51) D, with 70.93% and 44.19% of the eyes within ± 0.50 and ± 0.25 D, respectively. The mean difference between the noncycloplegic and cycloplegic subjective refraction using BWFOM was − 0.39 ± 0.46 (− 2.53 to 0.77) D, with 70.93% and 31.40% of the eyes within ± 0.50 and ± 0.25 D, respectively.

### Agreement test

Figure 2 illustrates the Bland–Altman plots with a 95% confidence interval (CI) of the limits of agreement between BWFOM and autorefraction and retinoscopy. The percentages of points falling outside the limits of agreement were 4.65% and 5.81% for BWFOM cycloplegic objective and subjective refraction, respectively.

Figure 3 illustrates the Bland–Altman plots with 95% CI of the limits of agreement between BWFOM noncycloplegic and cycloplegic refraction. The percentages of points falling outside the limits of agreement were 4.65% and 4.65% for objective and subjective refraction using the BWFOM, respectively.

### Examination time

The mean examination time was 264.88 ± 90.67 (121–440) s using BWFOM and 315.89 ± 95.31 (200–513) s using other refractive techniques. The BWFOM examination time was related to age (*P* < 0.001).

## Discussion

The new BWFOM utilizes wavefront aberration and adaptive optics technology, which are measured using a Hartmann–Shack wavefront sensor. A few devices have applied adaptive optics technology to refraction. Recent studies have shown that the Visual Adaptive Optics (VAO, Voptica S.L., Murcia, Spain) is a commercially available adaptive optics visual simulator with a Hartmann–Shack aberrometer [15]. It has been shown that with noncycloplegia, the subjective refraction of VAO was in good agreement with that of trial frame and trial ophthalmic lenses [15, 16]. This suggests that the use of adaptive optics technology in refraction is reliable and accurate, which was confirmed in our study. Another innovation of BWFOM is the ability to quickly obtain an optometric prescription with a spherical interval of 0.05 D. Our study is the first to apply this method to examine refractive errors in school-age children.

Several studies on children have shown that lack of cycloplegia is associated with a slight overestimation of myopia and marked errors in estimates of the prevalence of emmetropia and hyperopia [5]. In clinical practice, cycloplegic autorefraction and retinoscopy are recommended for children [17–19]. Consequently, we first compared BWFOM refraction with autorefraction and retinoscopy under cycloplegia to determine its accuracy for children’s refraction. We collected data from the right eyes of 86 participants whose mean age was 10.20 ± 1.99 (6–15) years. In our study, by comparing BWFOM objective refraction and autorefraction under cycloplegia, we found that the sphere, spherical equivalence, and the J45 of BWFOM were more biased toward negative. The mean differences were 0.11 D for sphere, 0.12 D for spherical equivalence, and 0.05 D for J45. Autorefraction was undercorrected, possibly because the 0.05-D spherical interval was more precise, and children were more realistically exposed to objective refraction under cycloplegia, while in clinical practice, we consider a refractive interval of 0.50 D to be significant. Therefore, this statistical difference is not clinically significant. The cylinder and J0 were not significantly different. Comparing the agreement between these two techniques, the mean difference for spherical equivalence was 0.12 D (95% CI, − 0.52 to 0.76 D), which is close to 0 D. These results suggest good agreement between BWFOM objective refraction and cycloplegic autorefraction. When compared with cycloplegic retinoscopy, the BWFOM subjective sphere was less myopic, and the cylinder and the J45 was more negative. The mean differences were − 0.08 D for the sphere, 0.15 D for the cylinder, and 0.04 D for the J45. The spherical equivalence and J0 were not significantly different. The sphere of retinoscopy in our study was overcorrected. However, Yi Z’s study found that the subjective spherical equivalence of the 0.05-D interval group was more negative in adults [12]. The difference may be due to the fact that children did not have as accurate cognitive feedback on nuances as adults according to different subjects, and secondly, the refraction method was different; we compared subjective refraction with the retinoscopy. In the agreement comparison, the mean difference for the spherical equivalence was − 0.01 D (95% CI, − 0.65 to 0.64), which is closer to 0 D. Thus, there is good agreement between BWFOM subjective refraction and retinoscopy. During our examinations, we found that BWFOM subjective refraction took less time, and the children were more cooperative. The total time cost using BWFOM refraction was significantly less than the total time of autorefraction and retinoscopy (264.88 ± 90.67 vs. 315.89 ± 95.31 s; *P* < 0.001), where the older child thereby spent less time. Traditional subjective refraction requires the professional optometrist to be patient, and the time-consuming process is difficult for most children [4, 20]. In populated and developing countries, refraction for children requires comprehensive consideration of accuracy, accessibility, and time. In conclusion, children’s refraction using the BWFOM agreed with autorefraction and retinoscopy with cycloplegia and performed faster and more conveniently.

We also compared BWFOM results before and after cycloplegia. For both BWFOM objective and subjective refraction, sphere and spherical equivalence with non-cycloplegia were significantly more myopic than those with cycloplegia. The mean differences were both − 0.37 D for sphere and spherical equivalence in objective refraction, which were − 0.40 D and − 0.39 D in subjective refraction, respectively. Several studies have shown that for different methods, noncycloplegic assessment overestimates myopia, even approximately 1 D [21–25]. However, our study showed that the difference was smaller, which may be due to the 0.05-D spherical interval, which made children adapt to more subtle optical changes; therefore, the true optometric results were reflected better using BWFOM. More samples are required to confirm these results. A good agreement was noted between refraction with and without cycloplegia. The mean differences for the spherical equivalence of objective and subjective refraction were − 0.37 D (95% CI, − 1.31 to 0.57) and − 0.39 D (95% CI, − 1.30 to 0.51), respectively. Therefore, both BWFOM subjective and objective refraction were more accurate after cycloplegia.

To date, researchers have considered a value of ≥ 0.50 D as a clinically significant difference in refraction [4, 26]. However, people are often sensitive to smaller dioptric changes. A study showed that 95% of patients were sensitive to dioptric changes under 0.25 D and that 44% of patients could distinguish between changes of < 0.125 D [11]. Yi Z et al. proved the resolution limit of the human eye to spherical lens change was about 0.05 D; different intervals of trial lens significantly influence the results of the refraction and duochrome test. The 0.05-D interval group produces a more accurate refraction and a better visual acuity, which is linked to a higher rate of red-green balance [12]. Consequently, more subtle changes can be perceived, and 0.50 D may no longer be a standard for determining whether it is clinically significant. Conversely, it has been pointed out that the importance of cycloplegia depends on the goal of the study. If a study is aimed at assessing the prevalence of myopia, cycloplegia is not necessary, despite the results that may be overestimated. Cycloplegia remains necessary for accurate refraction [5, 27]. Our study aimed to evaluate whether BWFOM can accurately measure pediatric refraction. BWFOM already provides the basis for accurate prescription due to the 0.05-D interval refraction and matching glasses. Therefore, for both the properties of BWFOM and future development of pediatric refraction, we recommend the cycloplegic refraction of BWFOM, which allows children to achieve greater precision in optical correction.

The limitations of this study include the relatively small sample size and the absence of age-segmented comparisons, which will be conducted in our future study.

## Conclusion

BWFOM is a new optometric device based on the principle of wavefront aberration and adaptive optics technology with built-in optical scales to perform both objective and subjective refraction in a bright environment. For children’s refractive errors, BWFOM is more convenient and quicker to obtain the proper prescription at a 0.05-D interval, and it is more accurate than autorefraction and retinoscopy under cycloplegia.

## Data Availability

Data and materials are available upon request from the corresponding author at doctxiaoyingwang@163.com.
